# Editorial: Women in obstetric and pediatric pharmacology: 2021

**DOI:** 10.3389/fphar.2023.1191285

**Published:** 2023-04-12

**Authors:** Catherine M. Sherwin, Yvonne S. Lin

**Affiliations:** ^1^ Department of Pediatrics, Wright State University Boonshoft School of Medicine/Dayton Children’s Hospital, Dayton, OH, United States; ^2^ University of Washington, Seattle, WA, United States

**Keywords:** pharmacology, pregnancy, pediatric, women in science, obstretic patients

## 1 Introduction

Women from all across the world have made extraordinary contributions to science throughout history. Names like Marie Curie, Ada Lovelace, and Rosalind Franklin are well-known (Rigby, 2023). However, other women are left out, losing their contributions to history. Less than 30% of researchers worldwide are women, according to UNESCO. Girls and women are discouraged from careers in science-related disciplines, notably in STEM research, due to ingrained biases and gender stereotypes (Charlesworth and Banaji, 2019; UNESCO UIS., 2019). Nonetheless, UNESCO has emphasized that in order to promote sustainable development, science, and gender equality are crucial. Gender equality must be promoted, misconceptions must be disproved, and girls and women should be encouraged to pursue STEM fields (Organisation for Economic Co-operation and Development OECD. 2023).

According to a substantial body of evidence, women are underrepresented in science, technology, engineering, and mathematics (STEM). Despite recent improvements, women still encounter considerable obstacles to entry and success in many industries. Generally, diversity and inclusion in STEM professions are becoming increasingly important, and numerous organizations are attempting to remove the obstacles that women and other underrepresented groups encounter (Fry et al., 2021).

Throughout history, women have made significant contributions to the science of pharmacology (Lavelle and Morris, 2020). We hope to spotlight some outstanding women in this research-themed Research Topic who are enhancing women’s and children’s health through wiser drug usage in both groups. We advocate for pharmacology-related research, instruction, and advocacy specifically focused on drug use in two groups: children and pregnant and nursing women. Clinical research and practice frequently fail to address the special pharmacological demands of these populations effectively, together with promoting the inclusion of infants and pregnant and nursing women in research trials (Liu and Mager, 2016).

Pregnancy, lactation, the unborn child’s development, and how drugs are absorbed, distributed, metabolized, and removed can all be significantly impacted by pregnancy and lactation. Studying the effectiveness and safety of drugs in these populations is crucial to ensuring their proper use (Burkey and Holmes, 2013; Feghali et al., 2015).

We are excited to introduce this Research Topic in the Frontiers in Pharmacology women in obstetric and pediatric pharmacology: 2021 series. A series that runs across Frontiers in Pharmacology. The research presented here demonstrates the variety of studies conducted across the entire spectrum of pharmacology research and presents developments in theory, experimentation, and methodology with applications to solve compelling problems.

## 2 Objective

Our goal was to gather and promote the contributions made by women across the globe and in the broad field of obstetric and pediatric pharmacology science and research.

## 3 Overview of contributions

### 3.1 Preeclampsia in a murine model

The main factor contributing to maternal and fetal morbidity is preeclampsia (PE). Endothelial and placental remodeling have been suggested as potential components of pregnancy evolution correction. Cytokines and growth factors, such as fibroblast growth factor 2, mediate this (FGF2). There is no research examining the effects of the administration of FGF2 during human pregnancy or in an animal model of PE, and its involvement in the development of PE is uncertain. Damage to the endothelium and podocytes in PE causes proteinuria. According to the study, rhFGF2 intravenously administered had positive and hypotensive effects. Moreover, giving rhFGF2 increased placental weight gain, fetal weight, and size in the rat PE-like model produced by L-NAME. These findings offer fresh perceptions of the function of FGF2 in modulating PE’s underlying pathogenic processes Martinez-Fierro et al.

We recognize the work of first author Margarita L Martinez-Fierro and the Mexican team who contributed to this study.

### 3.2 Adverse reactions in pediatric oncohematology

Adverse drug reactions (ADR) considerably increase morbidity and death in adults and children. Children are admitted to hospitals in between 2% and 5% of cases for ADR. The most commonly used chemotherapy medications were doxorubicin (24%) and 5-fluorouracil (20%). Cisplatin (44%) was also frequently used. With the help of these findings, the authors may pinpoint preventative measures to lower their incidence. Blood problems and infections were pegaspargase’s most common adverse drug reactions. ADRs for rituximab included infections and blood conditions. The study’s primary goal was to assess the prevalence and characteristics of suspected adverse drug reactions (ADR) to various drugs. Cancer-related medications are included as a risk factor for developing ADRs. Few pharmacovigilance studies, however, have evaluated ADR in juvenile oncohematological patients. These studies’ methodologies are incredibly diverse, which causes their outcomes to be quite unpredictable. For example, this study reported an ADR incidence of 72.2% (52 out of 72); however, the population was restricted to a list of particular medications, and the follow-up period was different Amaro-Hosey et al.

We recognize the work of the last author Antònia Agustí, and the team that worked on this study from Spain.

### 3.3 Severe bronchopulmonary dysplasia

Due to a lack of approved therapy, infants with severe bronchopulmonary dysplasia (BPD) are frequently treated with off-label medications. It is crucial to explain the exposure patterns in this population in order to identify the medications that need the most thorough efficacy and safety research. On the day of the study, diuretics were the most common class of medication given to patients. Babies receiving invasive ventilation were substantially more likely to be given diuretics than infants receiving non-invasive assistance (p 0.013). There is no FDA labeling to support the safety or efficacy of any drug use mentioned in this study, meaning they are all considered off-label. Given the paucity of research on the effectiveness of these medications in BPD, we might be subjecting infants to medications with both severe clinical damage and minimal therapeutic benefit. However, not much research has been done to determine the safety and effectiveness of infants with BPD. Therefore, there is no FDA labeling to support the safety or efficacy of any drug used disclosed in this study, which means they are all off-label Lewis et al.

We acknowledge the efforts of the US team that conducted this study, led by first author Tamorah Rea Lewis.

### 3.4 Drug self-medication in pregnancy

Self-medication is commonplace worldwide, with an estimated 32% of people using it. One of the vulnerable populations using the two types of self-medication is pregnant women; for instance, up to 10% of congenital impairments. Without a doctor’s prescription, they use medicines while pregnant, putting both the mother and the unborn at risk. That is a matter of public health. This study aimed to evaluate the most popular medications used, self-medication practices among pregnant women, and risk factors for these behaviors. The most common drug taken was paracetamol, and the most common symptoms described by pregnant women who self-medicated were nausea and vomiting. Self-medication was four times more common among women over 30 than among other groups. Health professionals can exert positive pressure through education and information on OTC drugs supplied during ANC. A medicine is considered safe if it is not known to be teratogenic, fetotoxic, or embryonic. Through monitoring and increasing awareness, prenatal surveillance specialists must play a part in reducing these dangers Chergaoui et al.

We acknowledge the contributions made by the Moroccan team that conducted this study, led by first author Samia Chergaoui.

### 3.4 Human placental drug transporters

Treatment discontinuation, disease relapse, and maternal morbidity may result from patients and healthcare professionals’ fear of fetal medication exposure. Even though placental drug transporters are essential for fetal exposure through active transport, most research only covers the third trimester, when most organogenesis has already occurred. Our goal was to identify changes in the protein activity, expression, and modifications of the five primary placental drug transporters, SERT, P-gp, NET, BCRP, and MRP3, dependent on gestational age (GA). Drug transporters in the placenta are essential for fetal exposure via active transport. Most research only covers the third trimester, when organogenesis is most advanced. Serotonin Transporter (SERT) and P-glycoprotein are crucial proteins involved in the placental transfer of those psychotropic drugs most frequently used during pregnancy (P-GP). The cleaved and uncleaved SERT subsets and GA showed a significant connection. There was no evidence of developmental regulation of NET levels. Throughout gestation, BCRP levels fell linearly. The results are not new; they support the technique concerning other relevant transporters. The authors present the most significant cohort with precise P-GP expression measurements throughout all three trimesters. It strengthens the mounting evidence that P.-gp plays a crucial protective role in the early stages of pregnancy when combined with earlier studies Goetzl et al.

We recognize the work of first author Laura Goetzl and the team that worked on this study from the USA.

### 3.5 Normal cervical tissue

With a high mortality rate for females worldwide, cervical cancer is now the fourth most prevalent type. A cornerstone of cervical cancer prevention programs is the early detection and treatment of cervical intraepithelial neoplasia (CIN) using a “see and treat” strategy. The restricted supply of N2O or CO2 gas for cryotherapy is a significant Research Topic. It is necessary to use an alternative therapeutic approach, such as the topical application of trichloroacetic acid (TCA). The epidermis and dermis are destroyed during TCA therapy due to necrosis. The breakdown of tissue occurs more quickly and profoundly at more significant concentrations. No studies examine the extent of tissue destruction caused by an 85% TCA solution on normal cervical tissue. Findings go beyond what CIN III can convey, which denotes depth in the cervical squamous epithelium. These findings lead the authors to conclude that the clinical and physical characteristics of the subjects’ cervix did not significantly influence the extent of tissue destruction caused by TCA in this study. As a result, this study can provide early information on the levels of damage caused by a single topical application of 85% TCA in normal cervical tissue, which are 1.16 and 1.01 mm in the anterior and posterior lips, respectively Nuranna et al.

We acknowledge the contribution made by the study's Indonesian team, led by first author Laila Nuranna.

### 3.6 Urinary tract infections

Urinary tract infections (UTIs) are a serious clinical Research Topic that frequently affects children and pregnant women. The main responsible bacteria include *Escherichia coli* and several other Gram-positive and Gram-negative bacteria. These patients frequently receive prescriptions for antimicrobial medications to treat UTIs. The efficacy of frequently used natural products such as cranberry juice/extracts, ascorbic acid, hyaluronic acid, probiotics, and multi-component formulations is discussed in this study. Asymptomatic bacteriuria is the presence of bacteria in the urine without any other signs of a UTI. In 0.5% of pregnant women, acute pyelonephritis develops. Bacteria’s release of endotoxins can bring on Anemia and uterine contractions that could result in preterm labor. Severe fever (>24 h) and high (>39 C (102.2 F) do seem to be more suggestive of UTIs. Amoxicillin and TMP-SMX have historically been used as first-line antibiotics in the US for pediatric UTIs. An alarming rise in resistance rates since 2009 was found, according to a recent study that examined the prevalence of antibiotic resistance among children with UTI over the previous 10 years. Examining complementary medicine options for kids with RUTIs may be beneficial due to the emergence of antibiotic-resistant urinary tract infections. However, some natural remedies can be used in both populations as a stand-alone treatment for UTIs and as a complement to antibiotics Hudson et al.

We acknowledge the contribution made by the team from the USA and Russia that worked on this project, led by the first author Rachel E. Hudson.

In conclusion, the study described here is a superb illustration of the contributions made by women to research and obstetric and pediatric pharmacology. Research Topic with a research focus, like this one, can be used to highlight women’s achievements in pharmacology, including doctors, scientists, trainees, and students. Additionally, there must be advocacy for the particular problems affecting women in research and their professional concerns in medicine and health. Finally, we acknowledge the contributions made by women to science across the world. Like most scientific disciplines, women have had difficulty rising to the top levels of pharmacology in business or academia.
